# Engineered *E. coli* W enables efficient 2,3-butanediol production from glucose and sugar beet molasses using defined minimal medium as economic basis

**DOI:** 10.1186/s12934-018-1038-0

**Published:** 2018-11-30

**Authors:** Anna Maria Erian, Martin Gibisch, Stefan Pflügl

**Affiliations:** Institute for Chemical, Environmental and Bioscience Engineering, Research Area Biochemical Engineering, Technische Universität Wien, Gumpendorfer Straße 1a, 1060 Vienna, Austria

**Keywords:** *E. coli* W, *pykA* knock-out, High rate and yield 2, 3-butanediol production, Sugar beet molasses, Chemically defined medium, Metabolic engineering, Promoter fine tuning, Acetoin, Complex protein hydrolysates

## Abstract

**Background:**

Efficient microbial production of chemicals is often hindered by the cytotoxicity of the products or by the pathogenicity of the host strains. Hence 2,3-butanediol, an important drop-in chemical, is an interesting alternative target molecule for microbial synthesis since it is non-cytotoxic. Metabolic engineering of non-pathogenic and industrially relevant microorganisms, such as *Escherichia coli*, have already yielded in promising 2,3-butanediol titers showing the potential of microbial synthesis of 2,3-butanediol. However, current microbial 2,3-butanediol production processes often rely on yeast extract as expensive additive, rendering these processes infeasible for industrial production.

**Results:**

The aim of this study was to develop an efficient 2,3-butanediol production process with *E. coli* operating on the premise of using cost-effective medium without complex supplements, considering second generation feedstocks. Different gene donors and promoter fine-tuning allowed for construction of a potent *E. coli* strain for the production of 2,3-butanediol as important drop-in chemical. Pulsed fed-batch cultivations of *E. coli* W using microaerobic conditions showed high diol productivity of 4.5 g l^−1^ h^−1^. Optimizing oxygen supply and elimination of acetoin and by-product formation improved the 2,3-butanediol titer to 68 g l^−1^, 76% of the theoretical maximum yield, however, at the expense of productivity. Sugar beet molasses was tested as a potential substrate for industrial production of chemicals. Pulsed fed-batch cultivations produced 56 g l^−1^ 2,3-butanediol, underlining the great potential of *E. coli* W as production organism for high value-added chemicals.

**Conclusion:**

A potent 2,3-butanediol producing *E. coli* strain was generated by considering promoter fine-tuning to balance cell fitness and production capacity. For the first time, 2,3-butanediol production was achieved with promising titer, rate and yield and no acetoin formation from glucose in pulsed fed-batch cultivations using chemically defined medium without complex hydrolysates. Furthermore, versatility of *E. coli* W as production host was demonstrated by efficiently converting sucrose from sugar beet molasses into 2,3-butanediol.

**Electronic supplementary material:**

The online version of this article (10.1186/s12934-018-1038-0) contains supplementary material, which is available to authorized users.

## Background

Microbial production of biofuels and chemicals gathers increasing interest in the light of scarce fossil fuels and consequently rising petroleum prices [1, 2]. Efficient production of bio-based fuels in amounts sufficient for downstream processing and subsequent industrial applications is, however, often hindered by the cytotoxicity of these chemicals [3]. 2,3-butanediol has been demonstrated as a promising, non-cytotoxic liquid fuel and bulk chemical for various applications including the use as food additive and antifreeze agent [4] or as precursor for the formation of methyl ethyl ketone [5]. The major drawback of natural 2,3-butanediol producers, for instance *Klebsiella oxytoca* [6], *Klebsiella pneumoniae* [7], *Serratia marcescens* [8], *Enterobacter aerogenes* [9] or *Enterobacter cloacae* [10], is that these organisms often require complex and expensive medium components (Table 1). Also the pathogenic nature of these strains limits the use as industrial production hosts [10, 11]. To overcome these issues, metabolic engineering of non-pathogenic microorganisms with minimal medium requirements and superior growth characteristics is favorable considering subsequent industrial applications.

**Table 1:** State-of-the-art of microbial 2,3-butanediol production

Organism	Strain	Over-expression	Feedstock	Operation mode	Titer [g l^−1^]	Yield [g g^−1^]	Productivity [g l^−1^ h^−1^]	Reference
*Enterobacter aerogenes*	KCTC 2190 *ΔldhA ΔscrR Δcra*	*scrAB* (*E. aerogenes*)	Sugar cane molasses	Fed-batch	140	0.39	2.59	[9]
*Enterobacter cloacae*	SDM *Δbdh ΔptsG Δldh ΔfrdA*	*bdh* (*B. pumilus*), *galP* (*E. cloacae SDM*)	Glucose + fructose + CSLP	Fed-batch	152	0.49	3.45	[10]
*Klebsiella oxytoca*	ME-UD-3 *aldA::Tcr*		Glucose	Fed-batch	130	0.48	1.63	[6]
*Klebsiella pneumonia*	SDM		Glucose + CSLP	Fed-batch	150	0.48	4.21	[7]
*Saccharomyces cerevisiae*	D452-2 *Δpdc1 Δpdc5*	*alsS* (*B. subtilis*), *alsD* (*B. subtilis*), *bdh1* (*S. cerevisiae*)	Glucose + YE + peptone	Fed-batch	96	0.28	0.39	[29]
*Serratia marcescens*	H30 *swrW*^−^		Sucrose + YE	Fed-batch	152	0.46	2.67	[8]
*Pichia pastoris*	X33	*alsS* (*B. subtilis*), *alsD* (*B. subtilis*)	Glucose + YE	Fed-batch	75	0.30	0.81	[30]
*Escherichia coli*	MG1655	*budA* (*K. pneumoniae*), *budB* (*K. pneumoniae*), *ydjL* (*B. subtilis*)	Glucose + YE	Batch	31	0.38	1.69	[31]
*Escherichia coli*	BL21(DE3)	*budA*, *budB* and *budC* (*E. cloacae* subsp*. dissolvens* SDM)	Glucose + YE	Fed-batch	74	0.41	1.19	[32]
*Escherichia coli*	W *ΔldhA ΔpflB ΔadhE ΔlpdA::K.p. lpdE354* *K Δmdh ΔarcA gltAR164L*	*ilvBN* (*E. coli*), *aldB* (*L. lactis* subsp*. lactis*), *bdh1* (*S. cerevisiae*)	Glucose + YE + tryptone	Fed-batch	88	0.35	1.87	[33]
*Escherichia coli*	JM109 *ΔldhA Δpta ΔadhE ΔpoxB*	*alsS* (*B. subtilis*, *alsD* (*B. subtilis*), *budC* (*K. pneumoniae*)	Glucose + YE	Shake flask	14.5	0.30	0.30	[34]
*Escherichia coli*	W	*budA*, *budB*, *budC* (*Enterobacter cloacae* subsp*. dissolvens*)	Glucose	Fed-batch High oxygen	52.1^a^	0.27^a^	4.53^a^	This study
*Escherichia coli*	W *ΔldhA ΔadhE Δpta ΔfrdA*	*budA*, *budB*, *budC* (*Enterobacter cloacae* subsp*. dissolvens*)	Glucose	Fed-batch Low oxygen	68.1	0.38	1.32	This study
*Escherichia coli*	W	*budA*, *budB*, *budC* (*Enterobacter cloacae* subsp*. dissolvens*)	Sugar beet molasses	Fed-batch	56.2	0.44	1.31	This study

Comparison of 2,3-butanediol titer, yield and productivity of natural and heterologous producers from different substrates

*YE* yeast extract, *CSLP* corn steep liquor powder

^a^Combined values for 2,3-butanediol and acetoin are given (ratio 2,3-butanediol:acetoin = 2.57)

One of the best-studied work-horses of the biotechnological industry meeting these requirements is *Escherichia coli* [12]*. E. coli* can grow in minimal culture medium and most industrially relevant strains utilize a broad range of carbon sources including pure sugars such as glucose, xylose and arabinose [13] as well as cheap industrial waste products such as acetate [14]. Moreover, a few strains, such as *E. coli* B-62, EC3123 or W [15–17], have been reported to be capable of utilizing sucrose which is a particularly interesting alternative carbon source due to its abundant availability in sugarcane and sugar beet molasses. Molasses as a by-product of sugar production contains large amounts of sucrose, a sugar directly fermentable by *E. coli.* Therefore, molasses has been reported to be economically more viable as a basis for production compared to corn-derived glucose [18]. Available reports of chemical production with *E. coli* from molasses are scarce and focus preliminary on the production of succinic acid or polyhydroxyalkanoates [19, 20]. In contrast, a plethora of studies are based on glucose as carbon source dealing with optimizing bioprocessing strategies and metabolic engineering approaches of *E. coli* for the microbial synthesis of diverse proteins [21–23] as well as biofuels [12, 24, 25]. Due to the extensive studies of *E. coli*, a broad range of genetic tools is available for insertions and modifications of metabolic pathways [26–28].

*Escherichia coli* does not natively produce 2,3-butanediol and hence requires three enzymes of the 2,3-butanediol pathway, i.e. α-acetolactate synthase (ALS), acetolactate decarboxylase (ALD) and butanediol dehydrogenase (BDH) for the conversion of precursor pyruvate to 2,3-butanediol [11]. Several studies show successful synthesis of l(+)-2,3-butanediol [35, 36], d(−)-2,3-butanediol [31, 37] or *meso*-2,3-butanediol [32, 34] with recombinant *E. coli* (Table 1). As a consensus of the collective previous work it can be stated that microaerobic conditions are beneficial for 2,3-butanediol synthesis in *E. coli*, as they are in natural producers, since 2,3-butanediol formation is coupled to the oxidation of NADH to NAD^+^.

Previously, the influence of the donor genes on 2,3-butanediol formation in *E. coli* has been described. In detail, the 2,3-butanediol operon from different natural producers was expressed. Using genes from *E. cloacae* subsp. *dissolvens* SDM and the native promoter of this operon allowed for production of up to 73.6 g l^−1^ 2,3-butanediol from glucose in complex medium containing yeast extract in fed-batch cultivations of *E. coli* BL21 after optimizing oxygen supply [32].

During the final stages of this study another paper was published using a somewhat similar approach. There, a mutation library from the native *nar* promoter of *E. coli* was constructed. Using this promoter library, fine-tuning of expression levels was used to optimize expression of the three pathway genes for 2,3-butanediol production. Fed-batch cultivations with the optimized construct under microaerobic conditions using glucose supplemented with yeast extract and peptone showed a titer of 88.0 g l^−1^ 2,3-butanediol [33].

Despite these significant advances, there is no information available how expression of individual genes from different donor organisms from individual promoters influences 2,3-butanediol productivity in different *E. coli* strain backgrounds.

Moreover, it should be noted that production of 2,3-butanediol in *E. coli* so far exclusively was achieved by addition of yeast extract or other complex nutrient mixtures [7, 9, 32–34, 38]. Since yeast extract is a complex mixture of diverse macro- and micronutrients [39] it influences and enhances microbial growth and potentially productivity and thus is commonly used for chemical production processes deployed at laboratory scale, e.g. for synthesis of ethanol [40], 1-butanol [41], isobutanol [42], isopropanol [43] or acetone [44]. However, yeast extract adds high costs to fermentation processes making it unattractive for industrial applications. The exact composition of nutrients contained in yeast extract may differ significantly between batches, making an evaluation of the impact of yeast extract on process performance challenging. Thus, there is a lack of information on how strain construction might be influenced in a system using defined minimal medium.

The aim of this study was to construct an efficient 2,3-butanediol producing strain of *E. coli* operating on the premise of using cost-effective chemically defined minimal medium. To achieve this goal, a library of expression vectors was constructed to study the influence of the expression level of individual genes from different organisms in vivo considering various strain backgrounds of *E. coli*. With a sound genetic construct and an *E. coli* strain capable to efficiently convert glucose into 2,3-butanediol, the system was further studied in pulsed fed-batch cultivations using defined minimal medium. This represents a novel approach as key process performance parameters titer, rate and yield of 2,3-butanediol synthesis have never been studied quantitatively in a system lacking complex hydrolysates as media additives. The performance of the defined 2,3-butanediol production system was further optimized with respect to oxygen supply and strain background in order to eliminate acetoin and by-product formation. Finally, transition towards industrial production was attempted by replacing the expensive and idealized model substrate glucose. Therefore, sugar beet molasses was for the first time considered and tested as basis for 2,3-butanediol production in *E. coli*, evaluating the potential of this valuable second-generation feedstock for microbial chemical synthesis.

## Results

### Strain construction and screening

The main aim of this study was to produce 2,3-butanediol using defined minimal medium or real substrates such as sugar beet molasses at high titer, rate and yield using recombinant *Escherichia coli*. In order to achieve this, a suitable expression strain was constructed. The influence of genes from different donor organisms, different promoter strengths for the expression of the individual genes and combination of genes from different organisms was investigated in *E. coli*. Special emphasis was given to find constructs that enable efficient production of 2,3-butanediol using chemically defined medium.

**Fig. 1:**
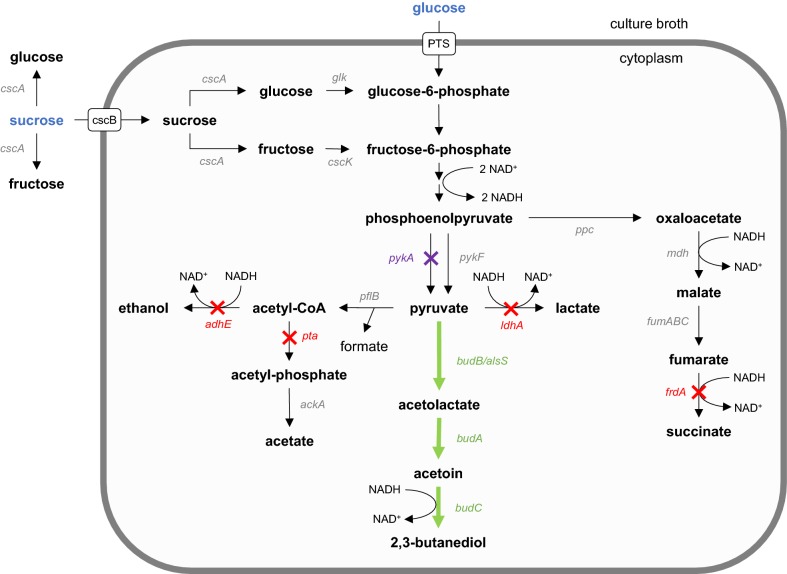
Simplified metabolic network of *Escherichia coli* for substrate uptake, mixed acid fermentation pathways and 2,3-butanediol production. Green arrows/genes represent the introduced heterologous 2,3-butanediol pathway. Red crosses/genes indicate genes knocked-out in *E. coli ∆ldhA ∆adhE ∆pta ∆frdA*/445_Ediss (445_Ediss ∆4) and the purple cross/gene indicates the additional knock-out in *E. coli ∆ldhA ∆adhE ∆pta ∆frdA ∆pykA*/445_Ediss (445_Ediss ∆5)

For construction of 2,3-butanediol biosynthetic pathways in *E. coli*, genes from three members of the family Enterobacteriaceae were chosen as gene donors for *budA*, *budB* and *budC* in this study, i.e. *Enterobacter cloacae* subsp*. dissolvens*, *Enterobacter cloacae* subsp*. cloacae* and *Klebsiella oxytoca.* Expression of these genes should enable production of 2,3-butanediol production in *E. coli* (Fig. 1). This approach was expanded by the expression of each gene from an individual constitutive promoter to enable fine-tuning of expression levels.

Initially, two different types of constructs per gene donor were generated with constitutive promoters of the Anderson promoter library, medium strength promoter BBa_J23114 (114p) and strong promoter BBa_J23105 (105p), arranging the genes in an order that reflects the occurrence of the respective enzymes in the metabolic pathway, i.e. *budB*-*budA*-*budC* (Fig. 1). The first type of construct included expression of all three genes using strong promoter 105p. In the second construct only *budC* was regulated by promoter 105p whereas *budB* and *budA* were controlled by medium strength promoter 114p based on the hypothesis that the conversion of acetoin to 2,3-butanediol is the rate-limiting step during 2,3-butanediol synthesis [31]. Additionally, constructs with a third constitutive promoter of the Anderson promoter library, weak promoter BBa_J23109 (109p), were constructed for further fine-tuning of the expression levels of the individual genes and to potentially reduce metabolic burden.

Upon successful assembly of all constructs using GoldenMOCS cloning [45], the construct library was tested in *E. coli* W. This strain was chosen due to its fast growth and high stress tolerance against environmental influences such as high acetate concentrations [15]. Shake flask cultivations of all *E. coli* W clones were conducted in triplicates to determine and compare 2,3-butanediol synthesis ability of all constructs. The screening was carried out using chemically defined minimal medium supplemented with 5% (w/v) glucose.

**Fig. 2:**
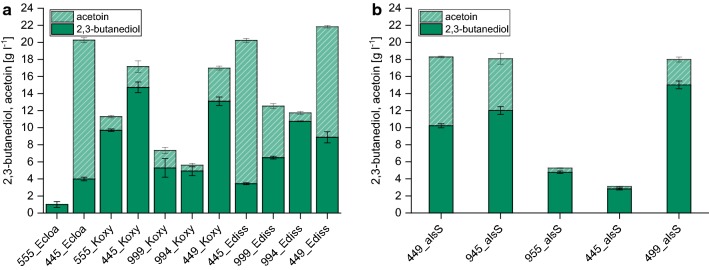
Screening result of the construct library for production of 2,3-butanediol in *E. coli* W after 48 h using 5% (w/v) glucose in minimal medium as substrate. Concentrations of 2,3-butanediol and acetoin obtained for the individual constructs are given as one bar (solid area = 2,3-butanediol, dashed area = acetoin). Designation of constructs as follows: Ecloa (*Enterobacter cloacae* subsp. *cloacae* DSM 30054), Koxy (*Klebsiella oxytoca* DSM 4798) and Ediss (*Enterobacter cloacae* subsp. *dissolvens* DSM 16657) represent gene donors for acetolactate synthase (*budB*), acetolactate decarboxylase (*budA*) and acetoin reductase/butanediol dehydrogenase (*budC*). *alsS* indicates that acetolactate synthase of *B. subtilis* was combined with *budA* and *budC* from Ediss. Three-digit numbers in front of donor organisms indicate promoters used for the three genes *budB*/*alsS*-*budA*-*budC* (5 = 105p, 4 = 114p and 9 = 109p)

As shown in Fig. 2a, all clones were able to synthesize 2,3-butanediol and acetoin in minimal medium. This represents a first proof-of-principle that production of 2,3-butanediol in a chemically defined background is possible using only glucose as the sole carbon source. Significant differences in diol titers (sum of 2,3-butanediol and acetoin) obtained for the different constructs were observed. The highest titers were obtained for three constructs able to accumulate more than 20 g l^−1^ diols, namely *E. coli* W expressing construct 114p_budB_114p_budA_105p_budC with genes from either *E. cloacae* subsp*. cloacae* (114p-114p-105p, 445_Ecloa) or *E. cloacae* subsp*. dissolvens* (445_Ediss) as well as 449_Ediss (20.3 ± 0.4, 20.2 ± 0.3 g l^−1^ and 21.8 ± 0.6 g l^−1^, respectively, given as mean and standard deviation) (Fig. 2a). The total diol yield Y_diol/S_ was 0.40 ± 0.01 g g^−1^, 0.40 ± 0.01 g g^−1^ and 0.41 ± 0.02 g g^−1^ for the three constructs, which corresponds to 80–82% of the maximum theoretical yield (Table 2).

**Table 2:** Screening result of the construct library for production of 2,3-butanediol in *E. coli* W after 48 h

Construct	Glucose [g l^−1^]	Biomass [g l^−1^]	Acetate [g l^−1^]	Y_diol/S_ [g g^−1^]	Y_X/S_ [g g^−1^]
555_Ecloa	13.21 ± 0.21	1.84 ± 0.09	5.65 ± 0.07	0.07 ± 0.02	0.14 ± 0.01
445_Ecloa	50.09 ± 0.01	4.19 ± 0.43	n.d.	0.40 ± 0.01	0.08 ± 0.01
555_Koxy	36.24 ± 1.08	2.65 ± 0.22	5.78 ± 0.23	0.31 ± 0.01	0.09 ± 0.03
445_Koxy	48.98 ± 1.93	3.22 ± 0.19	3.72 ± 0.24	0.35 ± 0.01	0.07 ± 0.01
999_Koxy	27.76 ± 1.70	2.68 ± 0.06	7.86 ± 1.20	0.26 ± 0.05	0.10 ± 0.01
994_Koxy	28.88 ± 1.35	2.78 ± 0.05	7.23 ± 0.51	0.20 ± 0.02	0.10 ± 0.01
449_Koxy	50.85 ± 0.54	4.69 ± 0.28	3.12 ± 0.11	0.33 ± 0.01	0.09 ± 0.01
445_Ediss	50.09 ± 0.01	4.29 ± 0.16	n.d.	0.40 ± 0.01	0.09 ± 0.01
999_Ediss	42.11 ± 0.91	3.99 ± 0.26	4.98 ± 0.20	0.30 ± 0.01	0.09 ± 0.01
994_Ediss	39.03 ± 0.31	3.90 ± 0.11	5.51 ± 0.39	0.30 ± 0.00	0.10 ± 0.01
449_Ediss	53.36 ± 0.16	4.57 ± 0.05	n.d.	0.41 ± 0.02	0.09 ± 0.01
449_alsS	52.82 ± 0.10	3.46 ± 0.11	0.47 ± 0.10	0.34 ± 0.01	0.07 ± 0.01
945_alsS	52.95 ± 0.19	3.16 ± 0.15	1.03 ± 0.03	0.34 ± 0.00	0.06 ± 0.01
955_alsS	24.93 ± 0.18	1.86 ± 0.03	5.15 ± 0.22	0.21 ± 0.01	0.07 ± 0.01
445_alsS	20.69 ± 0.16	1.98 ± 0.18	5.71 ± 0.38	0.15 ± 0.01	0.09 ± 0.01
499_alsS	52.68 ± 0.14	2.56 ± 0.06	1.81 ± 0.20	0.34 ± 0.01	0.05 ± 0.01

Consumed glucose, produced biomass and acetate as well as the diol yield (Y_diol/S_) and biomass yield (Y_X/S_) are shown. Designation of constructs: see Fig. 2

Results are shown as mean value of three replicates ± standard deviation. *n.d.* not detected

Compared to the three best performing constructs it was found that all other constructs containing either all strong promoters or two or three weak promoters produced significantly less diols (Fig. 2). Moreover, these strains showed incomplete glucose consumption. As an example, 555_Ecloa consumed less than 15 g l^−1^ glucose and it was observed that both very strong as well as weak over-expression of all three genes led to a strong drop of pH (data not shown) compared to the three best performing strains. This is likely linked to the formation of acetate, which was not observed for either 445_Ecloa, 445_Ediss or 449_Ediss, while all other constructs showed accumulation up to concentrations of 7.9 g l^−1^ (Table 2).

Expanding the construct library further, the effect of using genes from two different donor organisms to assemble the 2,3-butanediol biosynthetic pathway was studied. Therefore, *budA* and *budC* of *E. cloacae* subsp*. dissolvens* were combined with *alsS* of *Bacillus subtilis*. It has previously been shown that expression of *alsS* results in a tenfold higher acetolactate synthase activity in *E. coli* compared to the expression of *budB* [32], thus it was hypothesized that this way pyruvate could more efficiently be channeled into the 2,3-butanediol biosynthetic pathway. All constructs containing *alsS* instead of *budB* showed significantly lower diol production compared to 445_Ecloa, 445_Ediss and 449_Ediss, the best performing strains during the first stage of the construct screening (Fig. 2a, b). The highest yield obtained was 0.34 ± 0.01 g g^−1^ with construct 449_alsS and thus, is only 83% of the yield obtained with construct 449_Ediss. Construct 445_alsS displayed a sevenfold lower diol production compared to 449_Ediss (Fig. 2). Similar to constructs with three strong promoters to drive gene expression (555_Ecloa and 555_Koxy), also 445_alsS as well as all other constructs containing *alsS* instead of *budB* accumulated acetate (Table 2).

Collectively, a direct correlation between the obtained diol yield (per consumed substrate) and the total amount of substrate consumed was observed, where higher substrate consumption led to higher diol yield per consumed substrate (Additional file 1: Figure S1). In turn, constructs resulting in low diol yields and low overall substrate consumption displayed high acetate yields. Here, an indirect correlation between diol yield and acetate yield is observed (Additional file 1: Figure S2).

To further investigate this, some constructs showing low diol yields were reexamined in a system containing yeast extract [0.5% (w/v)]. The purpose of this screening was to evaluate if these constructs were generally unable to obtain high product titers and yields, or if low titers were a result of restricted nutrient availability in minimal medium. Four constructs were chosen that showed low product titers in minimal medium either due to strong overexpression, i.e. 555_Koxy, 555_Ecloa and 445_alsS, or weak overexpression, i.e. 999_Ediss, and were compared to a construct that resulted in both, high titers and yields, 445_Ediss.

As a consequence of adding yeast extract as a complex nutrient mixture to the culture medium, growth and product formation of all clones were enhanced (Table 3). Biomass yields between 0.10 ± 0.01 and 0.13 ± 0.01 g g^−1^ were obtained, which are slightly higher than biomass yields in minimal medium (Tables 2 and 3). Diol formation of 555_Ecloa and 445_alsS was increased 21- and 6.6-fold, respectively, in complex medium and diol titers of up to 21 g l^−1^ were achieved leading to high diol yields of 0.43 ± 0.01 g g^−1^. However, diol titers of 555_Koxy and 999_Ediss were similar to the ones in minimal medium and 555_Koxy accumulated 6.31 ± 0.26 g l^−1^ acetate whereas 999_Ediss produced 1.26 ± 0.05 g l^−1^ ethanol.

**Table 3:** Cultivation of five constructs in *E. coli* W using medium supplemented with 0.5% (w/v) yeast extract after 48 h

Construct	Glucose [g l^−1^]	Biomass [g l^−1^]	2,3-Butanediol [g l^−1^]	Acetoin [g l^−1^]	Acetate [g l^−1^]	Y_diol/S_ [g g^−1^]	Y_X/S_ [g g^−1^]
555_Ecloa	48.07 ± 0.09	5.04 ± 0.12	2.94 ± 0.04	17.51 ± 0.07	n.d.	0.43 ± 0.01	0.10 ± 0.01
555_Koxy	32.22 ± 4.82	3.62 ± 0.23	7.14 ± 1.53	2.42 ± 0.45	6.31 ± 0.26	0.29 ± 0.02	0.11 ± 0.01
445_alsS	47.64 ± 0.17	6.27 ± 0.25	1.94 ± 0.23	18.74 ± 0.63	n.d.	0.43 ± 0.01	0.13 ± 0.01
999_Ediss	47.52 ± 0.08	5.39 ± 0.16	4.03 ± 0.24	11.49 ± 0.43	n.d.	0.33 ± 0.01	0.11 ± 0.01
445_Ediss	47.72 ± 0.15	6.33 ± 0.07	4.27 ± 0.15	14.13 ± 0.40	n. d.	0.39 ± 0.01	0.13 ± 0.01

Consumed glucose, formed biomass and metabolites as well as the diol yield (Y_diol/S_) and biomass yield (Y_X/S_) are shown. Designation of constructs: see Fig. 2

Results are shown as mean value of three replicates ± standard deviation. *n.d.* not detected

These results indicate that choosing the right screening platform (i.e. yeast extract free) is crucial to obtain reliable results for further strain and process development.

As a result of the extensive strain construction and screening, several constructs capable of efficiently converting glucose in a chemically defined medium into 2,3-butanediol have been identified (445_Ecloa, 445_Ediss and 449_Ediss). Out of this pool, construct 445_Ediss was selected for further strain and process development. The reason for choosing 445_Ediss instead of 449_Ediss was that it was hypothesized that for longer processes such as fed-batch cultivations stronger expression levels of the last gene facilitating conversion of acetoin to 2,3-butanediol linked to NADH regeneration might be required.

In addition to the genetic construct, the influence of the strain background was investigated as another important aspect which could affect 2,3-butanediol synthesis in *E. coli*. Two commonly used *E. coli* strains offering distinct properties for chemical production, i.e. *E. coli* BL21(DE3) and *E. coli* K-12 MG1655 were tested with and without yeast extract for 2,3-butanediol production using the promising construct 445_Ediss. *E. coli* K-12 MG1655 produced diols both in chemically defined medium as well as in medium containing yeast extract, whereas *E. coli* BL21 (DE3) produced significant amounts of diols only in medium supplemented with yeast extract (Additional file 1: Table S1). The titers and yields obtained for both strains and media were lower compared to *E. coli* W in chemically defined medium, except for *E. coli* K-12 MG1655 cultivated in medium containing yeast extract, where comparable titer and yield were obtained (Additional file 1: Table S1). This emphasizes the importance of strain selection for successful production of chemicals in *E. coli* and confirmed *E. coli* W as a good production host for 2,3-butanediol production, potentially owing to its fast-growing nature and exceptional environmental stress resistance compared to common laboratory strains such as BL21 (DE3) and K-12 MG1655.

### Production of 2,3-butanediol in pulsed fed-batch cultivations

Following successful shake flask screenings, the most promising strain-construct combination *E. coli* W/445_Ediss (445_Ediss) was used as a sound basis for the development of an efficient 2,3-butanediol production process using glucose minimal-medium. To that end, pulsed fed-batch cultivations were used with the aim of this experiment being to identify key process parameters for efficient production of 2,3-butanediol. A 2-step cultivation strategy was chosen, where in the first aerobic step biomass was produced from 5% (w/v) glucose. Upon depletion of glucose from the culture medium, oxygen supply was switched to microaerobic conditions and the dissolved oxygen concentration was controlled at 0–1% by constantly adjusting the stirrer speed. The rationale behind this strategy was to allow for formation of a sufficient number of biocatalysts, and to subsequently provide cultivation conditions favoring formation of reduced products, i.e. connecting NADH/NAD^+^ recycling to product formation under microaerobic conditions (Fig. 1). During the microaerobic production phase, the culture was pulsed with a glucose-medium solution to restore a glucose concentration of 50 g l^−1^. This way, 445_Ediss was pulsed a total number of three times.

**Fig. 3:**
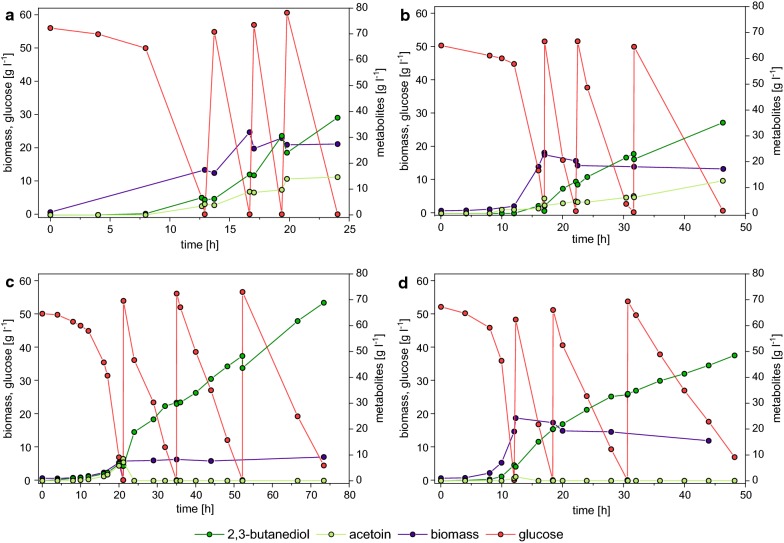
Two-step pulsed fed-batch cultivations in glucose minimal medium with an aerobic batch phase and a microaerobic production phase of *E. coli* W/445_Ediss under **a** high oxygen (DO 0–1%) and **b** low oxygen (constant stirrer and aeration). **c** *E. coli* W *∆ldhA ∆adhE ∆pta ∆frdA/*445_Ediss with low oxygen production phase and **d** *E. coli* W *∆ldhA ∆adhE ∆pta ∆frdA ∆pykA/*445_Ediss with low oxygen production phase. Glucose concentration was monitored throughout the cultivation in regular intervals. Once glucose was completely consumed, an appropriate amount of a glucose-medium solution was pulsed to the culture to restore a glucose concentration of 50 g l^−1^. Each cultivation was carried out in duplicates; only one cultivation is shown for better visualization (duplicate is shown in Additional file 2: Figure S3)

During the aerobic biomass formation phase, 445_Ediss reached biomass concentrations of 12.4 ± 1.3 g l^−1^ (Fig. 3a), while the biomass concentration averaged at 20.1 ± 0.2 g l^−1^ during the production phase (Table 4). On average, the rates for glucose uptake and diol formation were 17.0 ± 1.6 and 4.5 ± 0.3 g l^−1^ h^−1^ during the production phase, respectively (Table 4). At the end of the cultivation, a final diol titer of 52.1 ± 0.2 g l^−1^ was obtained. However, a significant portion of the total diol titer was comprised of acetoin, reflected in the ratio 2.57:1 of 2,3-butanediol:acetoin.

**Table 4:** Performance parameters of two-step pulsed fed-batch cultivations with *E. coli* W/445_Ediss (445_Ediss), *E. coli* W *∆ldhA ∆adhE ∆pta ∆frdA/*445_Ediss (445_Ediss ∆4) and *E. coli* W *∆ldhA ∆adhE ∆pta ∆frdA ∆pykA/*445_Ediss (445_Ediss ∆5) under high oxygen (DO 0–1%) and low oxygen (constant stirrer and aeration) supply

Strain	Oxygen supply	r_GLC_ [g l^−1^ h^−1^]	q_GLC_ [g g^−1^ h^−1^]	r_diol_ [g l^−1^ h^−1^]	q_diol_ [g g^−1^ h^−1^]	Y_diol/S_ [g g^−1^]	Y_by-product/S_ [g g^−1^]	Biomass [g l^−1^]	2,3-Butanediol [g l^−1^]	Acetoin [g l^−1^]	Diol Ratio
445_Ediss	High	16.99 ± 1.60	0.84 ± 0.07	4.53 ± 0.30	0.22 ± 0.01	0.27 ± 0.01	0.08 ± 0.02	20.14 ± 0.19	37.49 ± 0.18	14.59 ± 0.03	2.57
445_Ediss	Low	5.16 ± 0.13	0.38 ± 0.03	1.61 ± 0.04	0.12 ± 0.01	0.31 ± 0.02	0.22 ± 0.05	13.76 ± 1.28	34.32 ± 1.16	13.70 ± 1.53	2.53
445_Ediss ∆4	Low	3.51 ± 0.57	0.40 ± 0.08	1.32 ± 0.21	0.15 ± 0.03	0.38 ± 0.01	0.04 ± 0.01	8.78 ± 0.29	68.12 ± 1.08	0.13 ± 0.06	627.13
445_Ediss ∆5	Low	4.28 ± 0.27	0.25 ± 0.01	1.19 ± 0.06	0.07 ± 0.01	0.28 ± 0.01	0.04 ± 0.01	16.83 ± 0.15	48.50 ± 0.03	n.d.	n.a.

Volumetric and specific substrate uptake rates, r_GLC_ and q_GLC_, volumetric and specific diol (2,3-butanediol + acetoin) formation rates, r_diol_ and q_diol_, diol (2,3-butanediol + acetoin) and by-product yields, average biomass, 2,3-butanediol and acetoin concentrations and ratio 2,3-butanediol:acetoin (diol ratio) are given for the production phase of each cultivation. Values for individual pulses are given in Additional file 2: Table S2

Results are shown as mean value of two replicates ± standard deviation. *n.d.* not detected, *n.a.* not applicable

For efficient microbial conversion processes, the three key parameters titer, rate and yield need to be sufficiently high for a process to be competitive. While the diol productivity was 2.4-fold higher than the previously reported maximum [33] and a reasonable diol titer was obtained, the ratio 2,3-butanediol:acetoin and Y_diol/S_ was rather low. To evaluate whether the product yield and the ratio between 2,3-butanediol and acetoin can be improved by simply optimizing the aeration strategy, a 2-step cultivation with decreased oxygen supply during the production phase was carried out. Different from the culture with a dissolved oxygen of 0–1% both the stirrer speed and aeration rate were decreased at the end of the aerobic batch and kept constant throughout the production phase to ensure even lower levels of oxygen supplied to the culture. It was speculated that less oxygen supplied to the culture could increase both the total 2,3-butanediol titer and the product yield by increasing the amount of NADH available to the introduced heterologous 2,3-butanediol production pathway and simultaneously impairing the reverse reaction to acetoin.

Throughout the microaerobic production phase a DO level of 0% was observed. On average, the rates for glucose uptake and diol formation were only at 30 and 36%, respectively, of the values for the high oxygen cultivation (Table 4). At the end of the cultivation, a final diol titer of 48.0 ± 0.4 g l^−1^ was obtained (Fig. 3b). However, a significantly higher amount of by-products was formed during the cultivation with less oxygen supplied. In detail, a 2.7-fold increase of the combined titers of the mixed acid fermentation products acetate, formate, succinate, lactate and ethanol was observed (Additional file 2: Table S3). Despite more by-products formed, the overall Y_diol/S_ increased by 15% for the low oxygen cultivation compared to the high oxygen cultivation. Yet, the ratio of 2,3-butanediol:acetoin for both cultivations was comparable (2.53:1 vs. 2.57:1 for low and high oxygen, respectively) (Table 4).

To that end, construct 445_Ediss was introduced into *E. coli* W Δ*ldhA* Δ*adhE* Δ*pta* Δ*frdA* (445_Ediss Δ4). This strain background should in principle significantly reduce by-product formation due to deletions in genes responsible for the formation of the mixed acid fermentation products lactate (*ldhA*), acetate (*pta*) and succinate (*frdA*) and in addition ethanol (*adhE*) (Fig. 1). In turn, lower by-product formation should increase the pool of NADH available under low oxygen conditions, which might be beneficial for the overall titer of 2,3-butanediol as well as the ratio of 2,3-butanediol:acetoin.

Cultivated aerobically during the batch phase, a biomass concentration of 8.3 ± 0.3 g l^−1^ was observed, which represents a 2.1-fold lower Y_X/S_ for 445_Ediss Δ4 under aerobic conditions compared to 445_Ediss (18.1 ± 0.1 g l^−1^). On average, the rates for glucose uptake and diol formation further decreased compared to 445_Ediss cultivated under low oxygen conditions by 32% and 18%, respectively (Table 4). At the end of the cultivation, a titer of 68.1 ± 1.1 g l^−1^ was obtained, which is an increase of 42% compared to 445_Ediss (Fig. 3). As a result of the gene deletions in 445_Ediss Δ4, by-product formation decreased by 82% compared to 445_Ediss and in turn Y_diol/S_ increased by 22% (Table 4 and Additional file 2: Table S3). The most striking observation, however, was that the ratio 2,3-butanediol:acetoin was shifted almost completely towards 2,3-butanediol and virtually no acetoin was observed during the production phase (Fig. 3c).

As a sharp decrease in diol productivity was observed for the low oxygen cultivations (445_Ediss and 445_Ediss Δ4) in comparison to the high oxygen cultivation using 445_Ediss, another approach with the aim to improve glucose uptake and in turn 2,3-butanediol productivity was taken. Knock-out strains of *pykA* have been reported to increase glucose uptake in *E. coli* under anaerobic conditions leading to increased pyruvate-derived product formation [46]. Since *pykA* has been shown to be also active during microaerobic cultivations [47], it was hypothesized that knocking-out *pykA* in 445_Ediss Δ4 may also increase glucose consumption under microaerobic conditions and lead to higher 2,3-butanediol productivities. To investigate this hypothesis, *pykA* was deleted from the genome of *E. coli* W *∆ldhA ∆adhE ∆pta ∆frdA* using CRISPR/Cas9 (Fig. 1). Subsequently, low oxygen 2-step cultivations with *E. coli* W *∆ldhA ∆adhE ∆pta ∆frdA ∆pykA*/445_Ediss (445_Ediss Δ5) were performed.

At the end of the aerobic batch phase, a biomass concentration of 18.4 ± 0.3 g l^−1^ was obtained (Fig. 3d). This observation is quite remarkable, as it suggests that deletion of *pykA* restores the aerobic biomass yield of the deletion strain to a value comparable to that of *E. coli* W/445_Ediss (Y_X/S_ of 0.35 ± 0.01 g g^−1^ vs. 0.36 ± 0.01 g g^−1^). As hypothesized, the average glucose uptake rate during the batch and production phase increased by 95 and 22%, respectively, compared to 445_Ediss Δ4 cultivated under low oxygen conditions. However, the specific glucose uptake rate, i.e. the glucose uptake rate per biomass, of 445_Ediss Δ5 was only 63% of the rate observed for 445_Ediss and 445_Ediss Δ4 during the production phase and the specific diol formation rate was reduced by 50% compared to 445_Ediss Δ4 (Table 4). Similar to the cultivation with 445_Ediss Δ4, no acetoin was produced by 445_Ediss Δ5 and at the end of the third pulse a 2,3-butanediol titer of 48.5 ± 0.1 g l^−1^ was obtained (Fig. 3d). This represents a 29% decrease compared to 445_Ediss Δ4, accompanied by a 26% decrease of Y_diol/S_ (Table 4).

### Alternative substrates for 2,3-butanediol production

*Escherichia coli* can metabolize an array of carbon sources establishing the possibility to convert cheap and plentiful available renewable substrates, such as mannose, arabinose and xylose usually contained in hydrolysates of lignocellulosic biomass. Therefore, it was tested if *E. coli* W/445_Ediss is able to utilize these alternative carbon sources for 2,3-butanediol synthesis and if titers and yields are comparable to glucose-based cultivations. To that end, 445_Ediss was cultivated in shake flasks containing either 5% (w/v) mannose, arabinose or xylose as carbon source.

2,3-butanediol and acetoin could be produced from all three sugars. While Y_diol/S_ for mannose and arabinose was comparable to the value obtained for glucose, the diol yield for xylose was only 68% compared to glucose (Table 5). Similar to diol formation, also Y_X/S_ was reduced for xylose compared to glucose, and roughly only 50% of the sugar was consumed within 48 h (Table 5).

**Table 5:** Screening results for shake flask cultivations of *E. coli* W/445_Ediss after 48 h using 5% (w/v) carbon source in minimal medium

Substrate	Substrate [g l^−1^]	Biomass [g l^−1^]	2,3-Butanediol [g l^−1^]	Acetoin [g l^−1^]	Y_diol/S_ [g g^−1^]	Y_X/S_ [g g^−1^]
Glucose	50.09 ± 0.01	4.29 ± 0.16	3.44 ± 0.10	16.78 ± 0.24	0.40 ± 0.01	0.09 ± 0.01
Glucose + fructose	51.90 ± 0.08	5.56 ± 0.33	9.41 ± 1.76	10.92 ± 1.92	0.39 ± 0.01	0.11 ± 0.01
Fructose	51.30 ± 0.28	6.98 ± 0.50	4.57 ± 0.61	14.03 ± 0.70	0.36 ± 0.04	0.14 ± 0.01
Xylose	23.24 ± 2.00	3.01 ± 0.10	5.17 ± 0.87	1.11 ± 0.09	0.27 ± 0.02	0.06 ± 0.01
Arabinose	49.24 ± 0.28	5.04 ± 0.05	12.92 ± 0.02	5.65 ± 0.08	0.38 ± 0.01	0.10 ± 0.01
Mannose	46.25 ± 1.32	5.82 ± 0.02	2.56 ± 0.26	14.74 ± 0.29	0.37 ± 0.01	0.13 ± 0.01
Sucrose	38.23 ± 0.72	7.25 ± 0.16	4.51 ± 1.12	5.45 ± 1.55	0.26 ± 0.01	0.19 ± 0.01
Molasses	52.97 ± 0.24	10.89 ± 0.14	3.04 ± 0.94	15.31 ± 1.07	0.35 ± 0.01	0.21 ± 0.01

Consumed substrate, formed biomass and products as well as the diol yield (Y_diol/S_) and biomass yield (Y_X/S_) are shown

Results are shown as the mean value of three replicates ± standard deviation

Since cost-efficient resources are key to economic biotechnological processes, sugar beet molasses, an abundantly available side-product of sugar production, was evaluated as a potential substrate for 2,3-butanediol production with *E. coli.*

*Escherichia coli* W is one of the few strains capable of utilizing sucrose and its sucrose metabolism relies on either extracellular hydrolysis of sucrose to yield glucose and fructose (sucrose hydrolase, CscA) or on intracellular hydrolysis by CscA following the transport of sucrose into the cells (sucrose permease, CscB), phosphorylation of glucose (via glycolysis) and fructose (fructokinase, CscK) and subsequent degradation of both sugar phosphates via glycolysis [19, 48] (Fig. 1). Thus, a mixture of 2.5% (w/v) fructose + 2.5% (w/v) glucose, 5% (w/v) fructose and 5% (w/v) sucrose were tested in a preliminary experiment to compare glucose and sucrose utilization for 2,3-butanediol synthesis. Diol yields for fructose and fructose + glucose were comparable to the values using only glucose (Table 4). However, Y_X/S_ using fructose was 1.5-fold higher compared to glucose, while the yield was nearly unchanged for fructose + glucose. It was observed that glucose was preferentially consumed before fructose was taken up (data not shown) which is in accordance to the well-studied carbon catabolite repression in *E. coli* [49]. Cultivation of 445_Ediss with sucrose showed a reduction of the diol yield to 65% of the value obtained with glucose, possibly due to a significantly increased Y_X/S_ (2.1-fold increase compared to glucose, Table 5).

Based on the results from the experiments using pure sugars, sucrose from sugar beet molasses was evaluated for 2,3-butanediol production. Cultivation of 445_Ediss on molasses medium containing a sucrose concentration of 5% (w/v) led to the formation of 3.0 ± 0.9 g l^−1^ 2,3-butanediol and 15.3 ± 1.1 g l^−1^ acetoin which corresponds to a Y_diol/S_ of 0.35 ± 0.01 g g^−1^ (Table 5). Y_X/S_ was increased 2.3-fold in comparison to glucose but remained nearly unchanged compared to pure sucrose (Table 5).

**Fig. 4:**
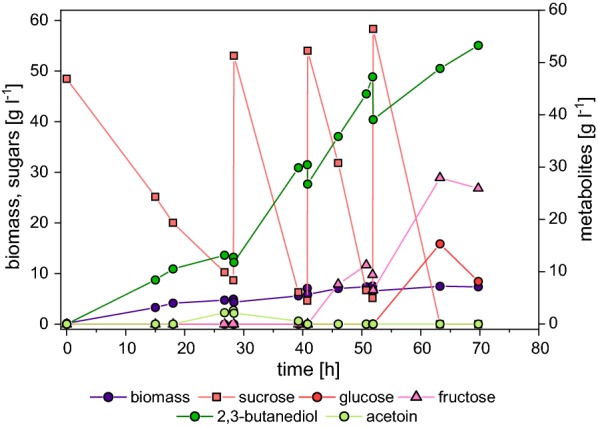
Small scale pulsed fed-batch cultivation of *E. coli* W/445_Ediss in molasses medium. Sucrose concentration was monitored throughout the cultivation in regular intervals. Once the sucrose concentration was < 10 g l^−1^ appropriate amounts of a molasses-medium solution were pulsed to the culture to restore a sucrose concentration of 50 g l^−1^. Cultivation was carried out in triplicates; only one cultivation is shown for better visualization (triplicate is shown in Additional file 2: Figure S3)

Based on these results, a pulsed fed-batch using 5% (w/v) molasses-derived sucrose in the batch and three molasses-medium pulses was used to investigate the growth and production characteristics of 445_Ediss using a real industrial substrate. The results obtained for this cultivation show that the use of sugar beet molasses in a two-step cultivation system is able to support efficient 2,3-butanediol production (Fig. 4 and Table 6). A titer of 56.3 ± 2.0 g l^−1^ 2,3-butanediol was obtained after three pulses and no acetoin formation was detected (Fig. 4), corresponding to a Y_diol/S_ during the production phase (i.e. three pulses) of 0.44 ± 0.03 g g^−1^ based on metabolized substrate. Furthermore, the productivity of the molasses-based system was comparable to the glucose-based system (1.31 g l^−1^ h^−1^ and 1.32 g l^−1^ h^−1^, respectively). However, it has to be noted that while during the first two pulses the rates for substrate uptake and 2,3-butanediol production were similar and peaked at 4.18 ± 0.49 g l^−1^ h^−1^ and 1.80 ± 0.07 g l^−1^ h^−1^, respectively, rates during the third pulse were significantly reduced and accumulation of fructose and glucose was observed (Fig. 4 and Table 6).

**Table 6:** Results from small-scale pulsed fed-batch cultivations of *E. coli* W/445_Ediss using molasses minimal medium

Pulse	r_S_ [g l^−1^ h^−1^]	q_S_ [g g^−1^ h^−1^]	r_diol_ [g l^−1^ h^−1^]	q_diol_ [g g^−1^ h^−1^]	Y_diol/S_ [g g^−1^]	Biomass [g l^−1^]
1st	3.90 ± 0.03	0.57 ± 0.01	1.54 ± 0.27	0.23 ± 0.04	0.39 ± 0.07	6.82 ± 0.08
2nd	4.18 ± 0.56	0.49 ± 0.05	1.80 ± 0.07	0.21 ± 0.02	0.44 ± 0.08	8.44 ± 0.32
3rd	1.77 ± 0.30	0.20 ± 0.03	0.88 ± 0.07	0.10 ± 0.01	0.50 ± 0.04	8.73 ± 0.32
Total	3.01 ± 0.22	0.38 ± 0.02	1.31 ± 0.06	0.16 ± 0.01	0.44 ± 0.03	7.99 ± 0.20

Volumetric and specific substrate uptake rates, r_S_ and q_S_, volumetric and specific diol (2,3-butanediol + acetoin) formation rates, r_diol_ and q_diol_, diol (2,3-butanediol + acetoin) yield and average biomass concentration are given individually for each pulse and for the total production phase

Results are given as mean value of three replicates ± standard deviation

## Discussion

The aim of this study was to generate a potent *Escherichia coli* strain for the microbial production of 2,3-butanediol under the premise of using cost-effective media. To that end, a sound genetic construct was created by comparison of four different donor organisms and promoter fine-tuning.

Using GoldenMOCS cloning, a library of expression vectors was generated, all of which enabled 2,3-butanediol production in *E. coli* W from glucose using chemically defined medium. Titer and yield varied greatly depending on which donor genes and which promoters were chosen for expression of the 2,3-butanediol pathway.

Expression of each gene of the pathway from solely strong promoters or weak promoters on a high copy number plasmid resulted in decreased 2,3-butanediol formation as well as accumulation of acetate and incomplete substrate consumption. When expressing all three genes from weak promoters, insufficient amounts of enzymes channelling carbon from the pyruvate node into the 2,3-butanediol production pathway might be responsible for the lower product titers in shake flasks. In case of using strong promoters, the microbial metabolism may be overburdened since excessive synthesis of enzymes often requires significant amounts of resources. Hence, insufficient levels of energy for cell maintenance and growth may be available [50] and consequently, this could result in an overall lower activity of the 2,3-butanediol pathway. This seems particularly true in case of cultivations using chemically defined minimal medium, where all components required for growth and maintenance need to be synthesized de novo, rather than relying on uptake of components from the culture medium in case yeast extract is supplied.

In both cases acetate was produced instead of 2,3-butanediol which was also observed for a vector control strain (data not shown). This could indicate an insufficient balance between the heterologously introduced 2,3-butanediol pathway on the one hand, and the natural metabolism of *E. coli* on the other hand. In the chemically defined system used for strain screening, this imbalance can also be seen as a direct correlation between the diol yield obtained and the amount of sugar consumed where a higher consumption rate of sugar corresponds to a higher diol yield. In addition, the product yield correlates indirectly with the acetate yield obtained. This can be explained by the fact that when balance between cell fitness and product formation is disturbed acetate accumulates as a consequence.

It was observed, that the expression of acetolactate synthase (*alsS*) from *B. subtilis* seemed to impose stress to a certain degree for all *alsS* expressing strains regardless of the promoter strength. This might be due to higher activity even at lower expression levels as *alsS* was reported to be tenfold more active in *E. coli* BL21 compared to other acetolactate synthases [32]. The fact that *alsS* expression from weaker promoters resulted in similar substrate consumption levels indicates that *alsS* has a higher activity also in *E. coli* W. Despite the higher activity, lower diol yields were observed in comparison to the best producing constructs (445_Ecloa, 445_Ediss and 449_Ediss). The reason for this observation is unknown but might be a consequence of stress imposed on the cell by expression of *alsS* in general and is in stark contrast to the hypothesis that higher acetolactate synthase activity should boost 2,3-butanediol synthesis. This indicates that high activity of an enzyme in a pathway alone is insufficient to support high yield production of 2,3-butanediol, but underlines that a balance between cell fitness and the activity of a production pathway must be achieved.

The rescreening of constructs in medium supplemented with yeast extract resulted in lower diol titers compared to the best performing constructs in chemically defined medium and showed that addition of yeast extract is sufficient to increase product yield in some cases (555_Ecloa and 445_alsS), while for other constructs (999_Ediss and 555_Koxy) no changes to chemically defined medium were observed. Therefore, it seems crucial to choose the screening system wisely, as addition of yeast extract would have resulted in identification of false positives for further strain and process development. However, this observation is in contrast to what has been previously reported where the promoter combination did not show a major influence on product concentration in shake flask cultivations carried out in LB medium supplemented with glucose [33].

Two-step bioreactor cultivations performed in this study showed that the production of 2,3-butanediol under defined conditions without addition of complex hydrolysates such as yeast extract is possible and only sufficient substrate supply is required to achieve high productivities. During these controlled fermentations, an inverse connection between product yield and productivity was observed. High oxygen supply led to high productivities and lower diol yields, while low oxygen supply increased diol yield at the cost of a lower productivity.

At high oxygen conditions, the highest 2,3-butanediol productivity ever reported for *E. coli* was obtained, which was 3.8-fold [32] and 2.4-fold [33] higher than rates reported in other studies. However, the low diol yield and poor 2,3-butanediol:acetoin ratio indicate non-optimal oxygen supply to the culture, resulting in insufficient amounts of NADH available for acetoin reduction to 2,3-butanediol.

Yet, rather than increasing product formation by narrowing down possibilities for the cell to regenerate NADH, decreased oxygen supply gave rise to by-product formation, as *E. coli* is well-known to produce mixed-acid fermentation products under low oxygen conditions or anaerobiosis. Forcing the cell to utilize 2,3-butanediol formation by taking away the possibility to regenerate NAD^+^ via the respiratory chain (by lower oxygen supply) or mixed acid fermentation pathways (by gene deletions) dramatically increased the diol ratio. The diol yield (0.38 g g^−1^) for the described chemically defined production system for 2,3-butanediol synthesis correlates nicely with previous reports using yeast extract (0.41 g g^−1^) [32] or yeast extract and peptone (0.35 g g^−1^) [33]. This is quite remarkable as *E. coli* in a defined system would have to dedicate more substrate to cell growth and maintenance compared to a complex system partly relying on extracellularly available precursors. Moreover, the volumetric productivity of the system was slightly higher to what has been reported by Xu et al. [32] and is 70% of the value reported by Hwang et al. [33] where the system was supplied with yeast extract and peptone.

In terms of productivity, an attempt was made to increase substrate uptake rate by deletion of pyruvate kinase II (*pykA*) of *E. coli*. The enzyme is reported to be active under both microaerobic and anaerobic conditions [47] and deletion of *pykA* was found to increase volumetric substrate uptake rates and productivities in *n*-butanol producing *E. coli* strains by increasing intracellular ATP levels [46]. However, introducing a *pykA* deletion in 445_Ediss ∆4 did not increase specific substrate uptake rates or diol productivity. Comparing these results to the findings of Zhao et al. [46] it seems that the observed effect is biomass-dependent. Deletion of *pykA* increased biomass concentration and in turn the volumetric substrate uptake rate but the specific substrate uptake rate (per biomass) was not positively affected by the deletion.

Although specific substrate uptake rates were not improved, the *pykA* deletion restored the biomass yield of the knock-out strain to values observed for *E. coli* W/445_Ediss, potentially indicating that indeed intracellular ATP levels were increased. This could be interesting for other applications where knock-out strains showing growth defects are required for production of target molecules in chemically defined medium lacking complex hydrolysates.

In this study, both high productivities (high oxygen) and high titer and yield (low oxygen) have been achieved. For industrial relevance, however, all three parameters need to be combined. Therefore, the results obtained here hold great promise, as the promising titer and yield that have been reached with 445_Ediss ∆4 could potentially be combined with high productivities by applying a continuous bioprocessing strategy. That way, the oxygen supply could be further optimized and the volumetric productivity of the 2,3-butanediol production system could be improved towards industrially relevant levels.

Upon successfully establishing a chemically defined production system for 2,3-butanediol, it was attempted to evaluate other sugar carbon sources than glucose for 2,3-butanediol production as well as to test the real industrial substrate sugar beet molasses for production.

2,3-butanediol production and biomass formation of *E. coli* W with comparable diol and biomass yields from various carbon sources was successfully demonstrated. Using sucrose from sugar beet molasses in a defined buffer system in small scale cultivations showed a higher diol yield compared to pure sucrose, which might be mediated by the presence of additional amino acids and trace elements in sugar beet molasses. As a direct result, less carbon from sucrose would need to be diverted for biomass formation.

Fed-batch cultivations using sucrose from non-pretreated sugar beet molasses resulted in an increased diol titer and yield compared to glucose as the substrate, while productivity was comparable for both substrates. The obtained titer (56 g l^−1^) was significantly lower than the titer of 154 g l^−1^ obtained with the natural producer *Enterobacter aerogenes* [9] (Table 1). However, the cultivation system used there was heavily supplemented with yeast extract and casamino acids, making direct comparison of the two systems difficult. Moreover, *E. aerogenes* is a human pathogen, which is a major drawback for any industrial production process.

During pulsed fed-batches using sugar beet molasses, lower substrate uptake rates and consequently production rates observed towards the end of the cultivation may be a consequence of enriching potentially inhibiting substances contained in molasses with each pulse as non-pretreated substrate was used. Accumulation of fructose and subsequently glucose was detected in the culture medium simultaneously to the declining substrate uptake and product formation rates. The accumulation of fructose starting during degradation of sucrose from the second pulse could be caused by insufficient activity of CscK which is responsible for intracellular phosphorylation of fructose. Deletion of *cscK* in *E. coli* W has been demonstrated to be responsible for fructose accumulation at high sucrose concentrations [48].

Concluding, these findings could for the first time show that 2,3-butanediol synthesis by *E. coli* W is possible using untreated sugar beet molasses as second generation feedstock and the obtained product yield is comparable to production from pure glucose. Moreover, these results indicate that molasses is both beneficial in terms of growth and product titers for *E. coli* W/445_Ediss in comparison to the utilization of pure glucose or sucrose, but also potentially inhibitory when used untreated.

## Conclusions

Using *E. coli* W, an efficient 2,3-butanediol production process was established without the requirement for addition of complex hydrolysates such as yeast extract or peptones. Strain construction was done with special emphasis on balancing cell fitness with production capacity by promoter fine-tuning and testing different strain backgrounds. The effect of yeast extract on screening results of a construct library in shake flasks was evaluated.

Two stage pulsed fed-batch cultivations were used to further study chemically defined production of 2,3-butanediol. By optimizing aeration and strain background, it was possible to show for the first time production of 2,3-butanediol with promising titer, rate and yield and no formation of acetoin as by-product from glucose in pulsed fed-batch cultivations using minimal medium without yeast extract. Continuous bioprocessing could be used in the future to increase 2,3-butanediol productivity while remaining high yield production by studying the effect of oxygen on 2,3-butanediol productivity in more detail. Furthermore, versatility of *E. coli* W as production host was demonstrated using an array of different carbon substrates, including conversion of sucrose from sugar beet molasses into 2,3-butanediol. Collectively, this study provides valuable information towards economic 2,3-butanediol production with *E. coli* W that might also prove useful for the synthesis of other chemicals.

## Materials and methods

### Bacterial strains and media

*Escherichia coli* BL21(DE3) (New England Biolabs, MA, USA) was used for all general cloning steps, for plasmid propagation and as production host. *E. coli* W (DSM 1116 = ATCC 9637, DSMZ, Braunschweig, Germany), *E. coli* W ∆*ldhA* ∆*adhE* ∆*pta* ∆*frdA* (kind gift of Prof. Michael Sauer, BOKU, Vienna, Austria) and *E. coli* W ∆*ldhA* ∆*adhE* ∆*pta* ∆*frdA* ∆*pykA* were used as production hosts.

All cloning steps, plasmid propagation steps, genome editing steps and precultures of *E. coli* were performed in liquid lysogeny broth (LB) containing soy peptone (10 g l^−1^), yeast extract (5 g l^−1^) and sodium chloride (10 g l^−1^) or on LB plates (LB containing 15 g l^−1^ agar).

Shake flask and batch cultivations were performed with chemically defined medium at pH 7 which was adapted from Riesenberg et al. [51] containing KH_2_PO_4_ (13.3 g l^−1^), (NH_4_)_2_HPO_4_ (4.0 g l^−1^), citric acid (1.7 g l^−1^), MgSO_4_ * 7H_2_O (1.2 g l^−1^), Fe(III)citrate (0.1 g l^−1^), EDTA (8.4 mg l^−1^), Zn(CH_3_COO)_2_ * 2 H_2_O (13.0 mg l^−1^), CoCl_2_ * 6 H_2_O (2.5 mg l^−1^), MnCl_2_ * 4 H_2_O (15.0 mg l^−1^), CuCl_2_ * 2 H_2_O (1.2 mg l^−1^), H_3_BO_3_ (3.0 mg l^−1^) and Na_2_MoO_4_ * 2 H_2_O (2.5 mg l^−1^). As carbon source, 5% (w/v) d-glucose, 5% (w/v) l-arabinose, 5% (w/v) d-fructose, 5% (w/v) d-xylose, 5% (w/v) sucrose, 5% (w/v) d-mannose or a mixture of 2.5% (w/v) d-glucose and 2.5% (w/v) d-fructose were used.

Feed medium for pulsed fed batch cultivations contained MgSO_4_ * 7H_2_O (1.2 g l^−1^), Fe(III)citrate (0.1 g l^−1^), EDTA (8.4 mg l^−1^), Zn(CH_3_COO)_2_ * 2 H_2_O (13.0 mg l^−1^), CoCl_2_ * 6 H_2_O (2.5 mg l^−1^), MnCl_2_ * 4 H_2_O (1.5 mg l^−1^), CuCl_2_ * 2 H_2_O (1.2 mg l^−1^), H_3_BO_3_ (3.0 mg l^−1^), Na_2_MoO_4_ * 2 H_2_O (2.5 mg l^−1^) and glucose (800 g l^−1^) or sucrose from sugar beet molasses (400 g l^−1^ sucrose) as required. An appropriate volume of feed medium was transferred to the culture to restore a concentration of 50 g l^−1^ glucose or sucrose once substrate was depleted.

50 µg ml^−1^ kanamycin or 100 µg ml^−1^ ampicillin were supplemented to liquid or solid media for all general cloning steps and cultivations as necessary.

### Plasmid and strain construction

GoldenMOCS [27, 45], a Golden Gate based cloning system, was used for all cloning steps in this study. All primers were purchased from Integrated DNA Technologies (IA, USA) and are listed in Additional file 3: Table S5.

The genes *budA*, *budB* and *budC* were PCR amplified from genomic DNA (obtained from DSMZ, Braunschweig, Germany) of *Klebsiella oxytoca* DSM 4798, *Enterobacter cloacae* subsp. *cloacae* DSM 30054 and *Enterobacter cloacae* subsp. *dissolvens* DSM 16657 using Q5 High-Fidelity DNA Polymerase (New England Biolabs, MA, USA). To remove restriction sites that would interfere with GoldenMOCS cloning, the genes were split in up to three individual parts and amplified using primer pairs part1_fw/part1_rev part2_fw/part2_rev and part3_fw/part3_rev as required. These parts were linked, and fusion sites were added by PCR using the respective primer pair budA_fw/budA_rev, budB_fw/budB_rev or budC_fw/budC_rev and equimolar amounts of the individual PCR fragments as template. Part 1 and 2 of *budB* from *E. cloacae* subsp. *cloacae* were directly joined during GoldenMOCS assembly. *alsS* from *Bacillus subtilis* was ordered from Integrated DNA Technologies (IA, USA).

The PCR fragments were assembled in BB1 of the GoldenMOCS as described in Sarkari et al. [45]. The clones were verified for correct PCR amplification and assembly via restriction digests and Sanger sequencing (Microsynth AG, Switzerland) using primers seq_fw and seq_rev (Additional file 3: Table S5).

To generate individual expression cassettes, each gene was assembled in BB2 with constitutive promoters BBa_J23109 (109p), BBa_J23114 (114p) or BBa_J23105 (105p) of the Anderson constitutive promoter library and BBa_B1001 as synthetic terminator. BB3 assembly was used to arrange the genes of one gene donor in a single expression vector and a construct library consisting of 16 different genetic constructs was generated (Additional file 3: Table S6). To check for correct assembly of all plasmids, restriction digests were carried out.

The no-SCAR protocol [52] relying on CRISPR/Cas9 and λ-Red mediated recombination was used to knock out *pykA* in *E. coli* W *∆ldhA ∆adhE ∆pta ∆frdA*. GoldenMOCS was used to exchange the spectinomycin resistance cassette of pKDsgRNA-ack (Addgene plasmid # 62654) with an ampicillin resistance, to alter the gRNA of pKDsgRNA-ack and to create a BB1 with a knock-out cassette consisting of two 500 bp homologous arms upstream and downstream of *pykA* of *E. coli* W fused together (BB1_HA_pykA). Plasmids pCas9-CR4 (Addgene plasmid # 62655) and pKDsgRNA-pykA were transformed into *E. coli* W *∆ldhA ∆adhE ∆pta ∆frdA*; the λ-Red genes were induced with 3.5% (w/v) L-arabinose at 37 °C, 200 rpm for 1 h; cells were electroporated with 1 µg of the PCR amplified knock-out cassette from BB1_HA_pykA and Cas9 was induced overnight at 30 °C with 100 µg l^−1^ anhydrotetracycline. The successful knock-out was confirmed by PCR and Sanger sequencing (Microsynth AG, Switzerland) and *E. coli* W *∆ldhA ∆adhE ∆pta ∆frdA ∆pykA* was cured of both plasmids.

### Pre-culture preparation

For *E. coli* pre-cultures, glycerol stocks, stored at -80 °C in 10% (w/v) glycerol, were streaked onto LB agar plates containing 50 µg ml^−1^ kanamycin and incubated overnight at 37 °C. A 1 l shake flask with 250 ml LB medium (50 µg ml^−1^ kanamycin) was inoculated with a single colony and incubated overnight at 37 °C and 200 rpm. The overnight cultures were harvested at an OD_600_ of ~ 4 and washed twice with 80 ml of 0.9% (w/v) NaCl solution (4800 rpm, 30 min, room temperature). The cells were resuspended in 20 ml 0.9% (w/v) NaCl and OD_600_ was determined. An appropriate volume of cell suspension was transferred to shake flasks (initial OD_600_ of 0.5) or bioreactors (initial OD_600_ of 1).

### Shake flask cultivations

Shake flask cultivation were carried out with a working volume of 100 ml in shake flasks with a total volume of 500 ml. An initial OD_600_ of 0.5 was used for all shake flask cultivations. Shake flasks were incubated at 37 °C and 200 rpm. Samples of *E. coli* cultivations were taken immediately after inoculation, after 24 h and after 48 h. In case of pulsed fed-batches, samples were taken before and after addition of molasses-medium solution to the culture.

### Bioreactor cultivations

Bioreactor cultivations were performed in four parallel DASGIP^®^ Benchtop Bioreactors for Microbiology or a DASbox^®^ Mini Bioreactor system (Eppendorf AG, Hamburg, Germany). The working volume was 1 l and 200 ml, respectively. All cultivations were carried out at 37 °C. The pH value was monitored by a pH electrode (Mettler-Toledo GmbH, Giessen, Germany) and was maintained at pH 7 by addition of NH_4_OH (12.5% v/v). During batch cultivations, aerobic conditions were maintained by changing stirrer speed, aeration rate and gas composition to maintain the dissolved oxygen above 30%, which was monitored by a VisiFerm DO 225 (Hamilton, Reno/NV, USA). Microaerobic conditions during production phases were achieved by either controlling the dissolved oxygen between 0 and 1% by changing stirrer speed and aeration rate (high oxygen cultivations) or by adjusting stirrer speed to 400 rpm and aeration to 1 vvm (= 60 l h^−1^) after the batch phase (low oxygen cultivations).

Samples were taken directly after inoculation, and at regular intervals during the batch phase. During production phase, samples were taken before and after addition of glucose-medium solution to the culture.

### Biomass determination and correlation with optical density

Cell dry weight of bioreactor cultivations was determined gravimetrically in triplicates. Therefore, 4 ml culture broth was centrifuged (4500 rpm, 10 min, 4 °C), washed with deionized water and dried in pre-weighed test glasses for at least 72 h at 105 °C.

A correlation between biomass and OD_600_ was established (biomass = OD_600_ × 0.452) and was used for the conversion of OD_600_ to biomass for all shake flask cultivations.

### HPLC analysis

Substrate and metabolite concentrations were measured by HPLC with an Ultimate 3000 system (Thermo Scientific, Waltham/MA, USA) using an Aminex HPX-87H column (300 × 7.8 mm, Bio-Rad, Hercules/CA, USA). The column was operated with 4 mM H_2_SO_4_ as mobile phase at 60 °C and a flow of 0.6 ml/min for 30 min. A refractive index detector (Refractomax 520, Thermo Scientific, Waltham/MA, USA) and an UV detector (Ultimate 3000, Thermo Scientific, Waltham/MA, USA) were used for peak detection. Controlling, monitoring and quantification of the HPLC run was performed with Chromeleon 7.2.6 Chromatography Data System (Thermo Scientific, Waltham/MA, USA). Samples and calibration standards were prepared by mixing 450 µl cell-free supernatant with 50 µl 40 mM H_2_SO_4_. 10 µl sample was injected for analysis and 5-point calibration curves were used for quantification.

### Enzymatic substrate quantification

For at-line measurements of glucose and sucrose during pulsed fed-batch cultivations, a Cedex Bio HT Analyzer (Roche, Switzerland) was used.

## Additional files

**Additional file 1: Figure S1.** Direct correlation between sugar uptake and diol yield. **Figure S2.** Indirect correlation between diol yield and acetate yield. **Table S1.** Screening result of E. coli strains BL21 (DE3) and K-12 MG1655 in chemically defined medium with and without yeast extract for production of 2,3-butanediol after 48 hours. Consumed glucose, produced biomass, 2,3-butanediol, acetoin and acetate as well as the diol yield (Y_diol/S_) and biomass yield (Y_X/S_) are shown.

**Additional file 2: Table S2.** Performance parameter for individual pulses of pulsed fed-batch cultivations. **Table S3.** By product formation during pulsed fed-batch cultivations. **Table S4.** C-mol yields and carbon recovery of two-step bioreactor cultivations (Fig. 3). **Figure S3.** Time course of duplicate two-step bioreactor cultivations (Fig. 3). **Figure S4.** Time course of triplicate small scale pulsed fed-batch cultivation of *E. coli* W/445_Ediss in molasses medium (Fig. 4).

**Additional file 3: Table S5.** List of used primers in this study. **Table S6:** List of plasmids and strains used in this study.
