# Supplementary material for: Engineered E. coli W enables efficient 2,3-butanediol production from glucose and sugar beet molasses using defined minimal medium as economic basis
Source: Microb Cell Fact. 2018 Nov 30;17:190. doi: 10.1186/s12934-018-1038-0 (PMC6267845; doi:10.1186/s12934-018-1038-0)
Supplement: Supplementary file 1 — Additional file 1: Figure S1. Direct correlation between sugar uptake and diol yield. Figure S2. Indirect correlation between diol yield and acetate yield. Table S1. Screening result of E. coli strains BL21 (DE3) and K-12 MG1655 in chemically defined medium with and without yeast extract for production of 2,3-butanediol after 48 hours. Consumed glucose, produced biomass, 2,3-butanediol, acetoin and acetate as well as the diol yield (Ydiol/S) and biomass yield (YX/S) are shown. [file 12934_2018_1038_MOESM1_ESM.docx]

**Additional File 1:**

**Figure S1**: Direct correlation between glucose consumption and diol yield (based on consumed substrate) for the constructs screened in Table 1 and Figure 1.

**Figure S2**: Indirect correlation between the diol yield (based on consumed substrate) and the acetate yield (based on consumed substrate) for the constructs screened in Table 1 and Figure 1.

**Table S1**: Screening result of E. coli strains BL21 (DE3) and K-12 MG1655 in chemically defined medium with and without yeast extract for production of 2,3-butanediol after 48 hours. Consumed glucose, produced biomass, 2,3-butanediol, acetoin and acetate as well as the diol yield (Y_diol/S_) and biomass yield (Y_X/S_) are shown.

| **Strain** | **yeast extract** | **glucose** | **biomass** | **2,3-butanediol** | **acetoin** | **acetate** | **Y_diol/S_** | **Y_X/S_** |
| --- | --- | --- | --- | --- | --- | --- | --- | --- |
|  | **[g l^-1^]** | **[g l^-1^]** | **[g l^-1^]** | **[g l^-1^]** | **[g l^-1^]** | **[g l^-1^]** | **[g g^-1^]** | **[g g^-1^]** |
| BL21 (DE3) | 5 | 49.99 ± 0.72 | 4.43 ± 0.26 | 13.07 ± 0.24 | 2.96 ± 0.03 | 5.99 ± 0.02 | 0.32 ± 0.01 | 0.09 ± 0.01 |
| BL21 (DE3) | 0 | 10.44 ± 0.22 | 1.75 ± 0.01 | 0.44 ± 0.10 | n.d. | 5.03 ± 0.05 | 0.04 ± 0.01 | 0.15 ± 0.01 |
| K12 MG1655 | 5 | 53.43 ± 0.13 | 4.70 ± 0.06 | 11.52 ± 0.22 | 10.29 ± 0.37 | 1.48 ± 0.13 | 0.41 ± 0.01 | 0.08 ± 0.01 |
| K12 MG1655 | 0 | 45.53 ± 0.73 | 4.84 ± 0.08 | 10.35 ± 0.10 | 2.30 ± 0.08 | 3.06 ± 0.06 | 0.28 ± 0.01 | 0.10 ± 0.01 |
