# Supplementary material for: Engineered E. coli W enables efficient 2,3-butanediol production from glucose and sugar beet molasses using defined minimal medium as economic basis
Source: Microb Cell Fact. 2018 Nov 30;17:190. doi: 10.1186/s12934-018-1038-0 (PMC6267845; doi:10.1186/s12934-018-1038-0)
Supplement: Supplementary file 2 — Additional file 2: Table S2. Performance parameter for individual pulses of pulsed fed-batch cultivations. Table S3. By product formation during pulsed fed-batch cultivations. Table S4. C-mol yields and carbon recovery of two-step bioreactor cultivations (Fig. 3). Figure S3. Time course of duplicate two-step bioreactor cultivations (Fig. 3). Figure S4. Time course of triplicate small scale pulsed fed-batch cultivation of E. coli W/445_Ediss in molasses medium (Fig. 4). [file 12934_2018_1038_MOESM2_ESM.docx]

**Additional File 2**

**Table S2**: Performance parameters of two-step pulsed fed-batch cultivations with *E. coli* W/445_Ediss (445_Ediss), *E. coli* W *∆ldhA ∆adhE ∆pta ∆frdA/*445_Ediss (445_Ediss ∆4) and *E. coli* W *∆ldhA ∆adhE ∆pta ∆frdA ∆pykA/*445_Ediss (445_Ediss ∆5) under high oxygen (DO 0 – 1 %) and low oxygen (constant stirrer and speed) supply. Volumetric and specific substrate uptake rates, r_GLC_ and q_GLC_, volumetric and specific product (2,3-butanediol +acetoin) formation rates, r_diol_ and q_diol_, and product (2,3-butanediol + acetoin) and by-product yields are given individually for each individual pulse.

| **Strain** | **Oxygen supply** | **Pulse** | **r_GLC_** | **q_GLC_** | **r_diol_** | **q_diol_** | **Y_diol/S_** | **Y_by-product/S_** |
| --- | --- | --- | --- | --- | --- | --- | --- | --- |
|  |  |  | **[g l^-1^ h^-1^]** | **[g g^-1^ h^-1^]** | **[g l^-1^ h^-1^]** | **[g g^-1^ h^-1^]** | **[g g^-1^]** | **[g g^-1^]** |
| 445_Ediss | High | 1^st^ | 16.94 ± 2.35 | 0.91 ± 0.13 | 4.57 ± 0.56 | 0.25 ± 0.03 | 0.27 ± 0.01 | 0.10 ± 0.01 |
|  |  | 2^nd^ | 22.39 ± 2.85 | 1.05 ± 0.13 | 6.43 ± 0.80 | 0.30 ± 0.04 | 0.29 ± 0.01 | 0.05 ± 0.01 |
|  |  | 3^rd^ | 13.88 ± 0.62 | 0.67 ± 0.01 | 3.40 ± 0.06 | 0.17 ± 0.01 | 0.25 ± 0.02 | 0.09 ± 0.01 |
| 445_Ediss | Low | 1^st^ | 9.82 ± 0.23 | 0.61 ± 0.01 | 2.13 ± 0.01 | 0.18 ± 0.06 | 0.22 ± 0.01 | 0.29 ± 0.01 |
|  |  | 2^nd^ | 5.46 ± 0.17 | 0.42 ± 0.03 | 1.62 ± 0.11 | 0.21 ± 0.15 | 0.30 ± 0.03 | 0.16 ± 0.10 |
|  |  | 3^rd^ | 3.35 ± 0.05 | 0.28 ± 0.04 | 1.43 ± 0.02 | 0.27 ± 0.23 | 0.43 ± 0.01 | 0.20 ± 0.03 |
| 445_Ediss ∆4 | Low | 1^st^ | 4.25 ± 0.49 | 0.50 ± 0.07 | 1.37 ± 0.16 | 0.16 ± 0.02 | 0.32 ± 0.01 | 0.06 ± 0.02 |
|  |  | 2^nd^ | 3.73 ± 0.65 | 0.43 ± 0.08 | 1.27 ± 0.26 | 0.15 ± 0.03 | 0.34 ± 0.01 | 0.04 ± 0.01 |
|  |  | 3^rd^ | 2.78 ± 0.45 | 0.31 ± 0.07 | 1.35 ± 0.21 | 0.15 ± 0.03 | 0.48 ± 0.01 | 0.02 ± 0.01 |
| 445_Ediss ∆5 | Low | 1^st^ | 8.49 ± 0.76 | 0.48 ± 0.05 | 2.34 ± 0.30 | 0.13 ± 0.02 | 0.28 ± 0.01 | 0.09 ± 0.01 |
|  |  | 2^nd^ | 4.30 ± 0.15 | 0.22 ± 0.01 | 1.12 ± 0.04 | 0.06 ± 0.01 | 0.26 ± 0.01 | 0.04 ± 0.01 |
|  |  | 3^rd^ | 2.81 ± 0.20 | 0.21 ± 0.01 | 0.85 ± 0.01 | 0.06 ± 0.01 | 0.30 ± 0.02 | n.a. |

n.a.: not applicable

**Table S3**: Product and by-product concentrations at the process end of two-step pulsed fed-batch cultivations with *E. coli* W/445_Ediss (445_Ediss), *E. coli* W *∆ldhA ∆adhE ∆pta ∆frdA/*445_Ediss (445_Ediss ∆4) and *E. coli* W *∆ldhA ∆adhE ∆pta ∆frdA ∆pykA/*445_Ediss (445_Ediss ∆5) under high oxygen (DO 0 – 1 %) and low oxygen (constant stirrer and speed).

| **Strain** | **Oxygen supply** | **2,3-butanediol** | **acetoin** | **biomass** | **acetate** | **ethanol** | **formate** | **lactate** | **succinate** |
| --- | --- | --- | --- | --- | --- | --- | --- | --- | --- |
|  |  | **[g l^-1^]** | **[g l^-1^]** | **[g l^-1^]** | **[g l^-1^]** | **[g l^-1^]** | **[g l^-1^]** | **[g l^-1^]** | **[g l^-1^]** |
| 445_Ediss | High | 37.49 ± 0.18 | 14.59 ± 0.03 | 20.78 ± 0.45 | 9.08 ± 0.61 | 1.45 ± 0.13 | 1.65 ± 0.01 | n.d. | 0.57 ± 0.01 |
| 445_Ediss | Low | 34.32 ± 1.16 | 13.70 ± 1.53 | 11.74 ± 2.01 | 0.90 ± 0.18 | 2.46 ± 0.12 | 5.94 ± 0.02 | 5.77 ± 0.29 | 9.97 ± 0.66 |
| 445_Ediss ∆4 | Low | 68.12 ± 1.08 | 0.13 ± 0.06 | 9.77 ± 0.87 | 3.25 ± 0.56 | 0.19 ± 0.02 | 2.14 ± 0.02 | n.d. | 0.64 ± 0.27 |
| 445_Ediss ∆5 | Low | 48.50 ± 0.03 | n.d. | 12.31 ± 0.70 | 2.88 ± 0.39 | 0.34 ± 0.01 | 1.82 ± 0.11 | n.d. | 1.19 ± 0.27 |

n.d.: not detected

**Table S4**: C-mol yields for biomass, diols, by-products, CO_2_ and residual glucose as well as carbon recovery for the two-step pulsed fed-batch cultivations (Fig. 3 and Table 4). Yields are given for the whole process including aerobic biomass formation and microaerobic production phase.

| **Strain** | **Oxygen supply** | **Y_X/S_** | **Y_diol/S_** | **Y_by-product/S_** | **Y_CO2/S_** | **Y_res GLC/S_** | **C-recovery** |
| --- | --- | --- | --- | --- | --- | --- | --- |
|  |  | **[Cmol Cmol^-1^]** | **[Cmol Cmol^-1^]** | **[Cmol Cmol^-1^]** | **[Cmol Cmol^-1^]** | **[Cmol Cmol^-1^]** | **[%]** |
| 445_Ediss | High | 0.15 ± 0.01 | 0.38 ± 0.01 | 0.07 ± 0.01 | 0.34 ± 0.06 | 0.08 ± 0.01 | 101.3 ± 8.2 |
| 445_Ediss | Low | 0.11 ± 0.02 | 0.41 ± 0.01 | 0.15 ± 0.01 | 0.23 ± 0.01 | 0.05 ± 0.01 | 94.8 ± 0.6 |
| 445_Ediss ∆4 | Low | 0.08 ± 0.01 | 0.56 ± 0.02 | 0.04 ± 0.01 | 0.24 ± 0.01 | 0.13 ± 0.04 | 104.1 ± 1.4 |
| 445_Ediss ∆5 | Low | 0.16 ± 0.01 | 0.40 ± 0.01 | 0.04 ± 0.01 | 0.24 ± 0.01 | 0.10 ± 0.01 | 93.0 ± 3.9 |

**Figure S3**: Time course of the duplicate two-step pulsed fed-batch cultivations in glucose minimal medium with an aerobic batch phase and a microaerobic production phase of *E. coli* W/445_Ediss under (a) high oxygen (DO 0 - 1 %) and (b) low oxygen (constant stirrer and aeration). (c) *E. coli* W ∆*ldhA* ∆*adhE* ∆*pta* ∆*frdA*/445_Ediss with low oxygen production phase and (d) *E. coli* W ∆*ldhA* ∆*adhE* ∆*pta* ∆*frdA* ∆*pykA*/445_Ediss with low oxygen production phase.

**Figure S4:** Time course of the triplicate small scale fed-batch cultivations in sugar beet molasses minimal medium of *E. coli* W/445_Ediss.
