# Supplementary material for: Engineered E. coli W enables efficient 2,3-butanediol production from glucose and sugar beet molasses using defined minimal medium as economic basis
Source: Microb Cell Fact. 2018 Nov 30;17:190. doi: 10.1186/s12934-018-1038-0 (PMC6267845; doi:10.1186/s12934-018-1038-0)
Supplement: Supplementary file 3 — Additional file 3: Table S5. List of used primers in this study. Table S6: List of plasmids and strains used in this study. [file 12934_2018_1038_MOESM3_ESM.docx]

**Table S5:** List of used primers in this work.

| **Name** | **Sequence (5' - 3')** |
| --- | --- |
| Koxy_budA_fw | GATCGGTCTCACatgAACCATTCTGCTGAATGC |
| Koxy_budA_rev | GATCGGTCTCAAAGCTTAGTTTTCGACTGAGCGAATG |
| Koxy_budB_part1_fw | CTGTTCTGCGCCCTG |
| Koxy_budB_part1_rev | GCAGCGAATCGAAACTTTATC |
| Koxy_budB_part2_fw | GATAAAGTTTTCGATTCGCTGC |
| Koxy_budB_part2_rev | GGCTGTCTCCAGCTC |
| Koxy_budB_part3_fw | GAGCTGGAGACAGCC |
| Koxy_budB_part3_rev | GATCGGTCTCAAAGCTCAAAAGTATTTGACTGAGATGAAGC |
| Koxy_budB_fw | GATCGGTCTCACatgGATAATCAACATCAACCGC |
| Koxy_budB_rev | GATCGGTCTCAAAGCTCAAAAGTATTTGACTGAGATGAAGC |
| Koxy_budC_part1_fw | GATCGGTCTCACatgAAAAAAGTCGCACTCGTG |
| Koxy_budC_part1_rev | GCGACATCTTCCGGC |
| Koxy_budC_part2_fw | GCCGGAAGATGTCGC |
| Koxy_budC_part2_rev | GTCACCGCTCTGGATAATG |
| Koxy_budC_fw | GATCGGTCTCACatgAAAAAAGTCGCACTCGTG |
| Koxy_budC_rev | GATCGGTCTCAAAGCTTAGCTAAATACCATGCCGCC |
| Ecloa_budA_part1_fw | GCCAATCGACAGGTTACC |
| Ecloa_budA_part1_rev | CGCAGTGTCTCGCATAAAC |
| Ecloa_budA_part2_fw | GTTTATGCGAGACACTGCG |
| Ecloa_budA_part2_rev | GATCGGTCTCAAAGCTTAGTTTTCGACGGAACGG |
| Ecloa_budA_fw | GATCGGTCTCACatgATGCACTCATCTGCC |
| Ecloa_budA_rev | GATCGGTCTCAAAGCTTAGTTTTCGACGGAACGG |
| Ecloa_budB_part1_fw | GATCGGTCTCACatgAACAGTGAGAAACAGTCAC |
| Ecloa_budB_part1_rev | GATCGGTCTCAGGACACCGATACCACTTTG |
| Ecloa_budB_part2_fw | GATCGGTCTCAGTCCGGCGACGG |
| Ecloa_budB_part2_rev | GATCGGTCTCAAAGCTCACAAAATCTGGCTGAGATG |
| Ecloa_budC_fw | GATCGGTCTCACatgCAAAAAGTTGCTCTCGTAAC |
| Ecloa_budC_rev | GATCGGTCTCAAAGCTTAGTTGAACACCATCCCAC |
| Ediss_budA_part1_fw | GCCAATCGACAGGTTACC |
| Ediss_budA_part1_rev | CCTCACAGTCGCAG |
| Ediss_budA_part2_fw | CTGCGACTGTGAGGC |
| Ediss_budA_part2_rev | GATCGGTCTCAAAGCTTAGTTTTCGACGGAACGG |
| Ecloa_budA_fw | GATCGGTCTCACatgATGCACTCATCTGCC |
| Ediss_budA_rev | GATCGGTCTCAAAGCTTAGTTTTCGACGGAACGG |
| Ediss_budB_part1_fw | CCTTGAATCGACGCTG |
| Ediss_budB_part1_rev | CAGCGTCGATTCAAGG |
| Ediss_budB_part2_fw | CAGCGTCGATTCAAGG |
| Ediss_budB_part2_rev | GAGCAGGCGTTGATG |
| Ediss_budB_fw | GATCGGTCTCACatgAACAGTGAGAAACAGTCAC |
| Ediss_budB_rev | GATCGGTCTCAAAGCTCACAAAATCTGGCTGAGATG |
| Ediss_budC_fw | GATCGGTCTCACatgCAAAAAGTTGCTCTCGTAAC |
| Ediss_budC_rev | GATCGGTCTCAAAGCTTAGTTGAACACCATCCCAC |
| seq_fw | GCAGTCCAGTTACGCTG |
| seq_rev | CGTGGACCGATCATACG |

**Table S6**: List of used plasmids and strains in this work.

| **Name** | **Acronym** | **Source** |
| --- | --- | --- |
| **Plasmids** |  |  |
| *BB1_pIDTSmart(Kan^R^)* |  |  |
| _FS1_105p_FS2 |  | Sarkari et al (2017) |
| _FS1_109p_FS2 |  | Sarkari et al (2017) |
| _FS1_114p_FS2 |  | Sarkari et al (2017) |
| _FS1_TT_FS2 |  | Sarkari et al (2017) |
| _FS2_alsS_Bsub_FS3 |  | This work |
| _FS2_amilCP_FS3 |  | Sarkari et al (2017) |
| _FS2_budA_Koxy_FS3 |  | This work |
| _FS2_budB_Koxy_FS3 |  | This work |
| _FS2_budC_Koxy_FS3 |  | This work |
| _FS2_budA_Ediss_FS3 |  | This work |
| _FS2_budB_Ediss_FS3 |  | This work |
| _FS2_budC_Ediss_FS3 |  | This work |
| _FS2_budA_Ecloa_FS3 |  | This work |
| _FS2_budB_Ecloa_FS3 |  | This work |
| _FS2_budC_Ecloa_FS3 |  | This work |
| *BB2_pUC(Amp^R^)* |  |  |
| _LinkerA_FS1_FS4_LinkerB |  | Sarkari et al (2017) |
| _LinkerB_FS1_FS4_LinkerC |  | Sarkari et al (2017) |
| _LinkerC_FS1_FS4_LinkerD |  | Sarkari et al (2017) |
| _LinkerA_105p_budB_Ecloa_TT_LinkerB |  | This work |
| _LinkerA_105p_budB_Ediss_TT_LinkerB |  | This work |
| _LinkerA_105p_budB_Koxy_TT_LinkerB |  | This work |
| _LinkerA_114p_alsS_Bsub_TT_LinkerB |  | This work |
| _LinkerA_114p_budB_Ecloa_TT_LinkerB |  | This work |
| _LinkerA_114p_budB_Ediss_TT_LinkerB |  | This work |
| _LinkerA_114p_budB_Koxy_TT_LinkerB |  | This work |
| _LinkerA_109p_alsS_Bsub_TT_LinkerB |  | This work |
| _LinkerA_109p_budB_Ediss_TT_LinkerB |  | This work |
| _LinkerA_109p_budB_Koxy_TT_LinkerB |  | This work |
| _LinkerB_105p_budA_Ecloa_TT_LinkerC |  | This work |
| _LinkerB_105p_budA_Ediss_TT_LinkerC |  | This work |
| _LinkerB_105p_budA_Koxy_TT_LinkerC |  | This work |
| _LinkerB_109p_budA_Ediss_TT_LinkerC |  | This work |
| _LinkerB_109p_budA_Koxy_TT_LinkerC |  | This work |
| _LinkerB_114p_budA_Ecloa_TT_LinkerC |  | This work |
| _LinkerB_114p_budA_Ediss_TT_LinkerC |  | This work |
| _LinkerB_114p_budA_Koxy_TT_LinkerC |  | This work |
| _LinkerC_105p_budC_Ecloa_TT_LinkerD |  | This work |
| _LinkerC_105p_budC_Ediss_TT_LinkerD |  | This work |
| _LinkerC_105p_budC_Koxy_TT_LinkerD |  | This work |
| _LinkerC_109p_budC_Ediss_TT_LinkerD |  | This work |
| _LinkerC_109p_budC_Koxy_TT_LinkerD |  | This work |
| *BB3_pUC(Kan^R^)* |  |  |
| _LinkerAD |  | Sarkari et al (2017) |
| _LinkerA_109p_alsS_105p_budA_105p_budC_TT_Ediss_LinkerD | 955_alsS | This work |
| _LinkerA_109p_alsS_114p_budA_105p_budC_TT_Ediss_LinkerD | 945_alsS | This work |
| _LinkerA_114p_alsS_109p_budA_109p_budC_TT_Ediss_LinkerD | 499_alsS | This work |
| _LinkerA_114p_alsS_114p_budA_105p_budC_TT_Ediss_LinkerD | 445_alsS | This work |
| _LinkerA_114p_alsS_114p_budA_109p_budC_TT_Ediss_LinkerD | 449_alsS | This work |
| _LinkerA_105p_budB_105p_budA_105p_budC_TT_Ecloa_LinkerD | 555_Ecloa | This work |
| _LinkerA_114p_budB_114p_budA_105p_budC_TT_Ecloa_LinkerD | 445_Ecloa | This work |
| _LinkerA_109p_budB_109p_budA_109p_budC_TT_Ediss_LinkerD | 999_Ediss | This work |
| _LinkerA_109p_budB_109p_budA_114p_budC_TT_Ediss_LinkerD | 994_Ediss | This work |
| _LinkerA_114p_budB_114p_budA_105p_budC_TT_Ediss_LinkerD | 445_Ediss | This work |
| _LinkerA_114p_budB_114p_budA_109p_budC_TT_Ediss_LinkerD | 449_Ediss | This work |
| _LinkerA_105p_budB_105p_budA_105p_budC_TT_Koxy_LinkerD | 555_Koxy | This work |
| _LinkerA_109p_budB_109p_budA_109p_budC_TT_Koxy_LinkerD | 999_Koxy | This work |
| _LinkerA_109p_budB_109p_budA_114p_budC_TT_Koxy_LinkerD | 994_Koxy | This work |
| _LinkerA_114p_budB_114p_budA_105p_budC_TT_Koxy_LinkerD | 445_Koxy | This work |
| _LinkerA_114p_budB_114p_budA_109p_budC_TT_Koxy_LinkerD | 449_Koxy | This work |
|  |  |  |
| **Strains** |  |  |
| *Escherichia coli* BL21(DE3) |  | NEB |
| *Escherichia coli* K-12 MG1655 DSM 18039 |  | DSMZ |
| *Escherichia* *coli* W DSM 1116 (=ATCC 9637) |  | DSMZ |
| *Escherichia coli* W *∆ldhA ∆adhE ∆pta ∆frdA* |  | University of Natural Resources and Life Sciences, BOKU, Vienna, Austria |
| *Escherichia coli* W *∆ldhA ∆adhE ∆pta ∆frdA ∆pykA* |  | This work |

BB: backbone; FS: fusion site; 105p, 109p, 114p: Promoters BBa_J23105, BBa_J23109 and BBa_J23114 of the Anderson promoter library; TT: artificial promoter BBa_B1001; Bsub: *Bacillus subtilis;* Ecloa: *Enterobacter cloacae* subsp. *cloacae;* Ediss: *Enterobacter cloacae* subsp. *dissolvens;* Koxy: *Klebsiella oxytoca*
